# Early intervention in psychotic disorders: Challenges and relevance in the Indian context

**DOI:** 10.4103/0019-5545.69228

**Published:** 2010-01

**Authors:** Matcheri S. Keshavan, Amresh Shrivastava, Bangalore N. Gangadhar

**Affiliations:** Department of Psychiatry, Harvard Medical School, Boston MA; 1 Lawson Health Research Institute, Consultant, Early Intervention of Psychosis Program, Regional Mental Health Care. Department of Psychiatry, The University of Western Ontario. London, Ontario, Canada; 2 National Institute of Mental Health and Neurosciences, Bangalore, India

**Keywords:** Early intervention, India, neurobiology, psychosis, schizophrenia

## Abstract

Over the last two decades, there has been an increasing interest in the concept of early intervention (EI) in psychotic disorders, notably schizophrenia. Several lines of research underlie this emerging paradigm shift: (a) an increasingly well-established association between the duration of prolonged untreated illness and poor outcome; (b) evidence of progressive neurobiological changes in the early course of schizophrenia both in the pre-psychotic and psychotic phases, as evidenced by brain imaging studies in schizophrenia; and (c) emerging data, albeit preliminary, suggesting the efficacy and effectiveness of EI programs in improving the outcome in these patients. Mental health service systems across the globe, including Asian countries, have been incorporating specialized early intervention programs. However, literature on EI in the Indian setting is relatively sparse. In this article, we will review the rationale and approaches to EI and the application of these approaches to the Indian context, in light of the available literature. We also examine the constraints in the implementation of EI. Controlled data are needed to evaluate EI and the roadblocks to them, in order to implement EI in the resource-strapped mental health service settings in India.

## INTRODUCTION

Schizophrenia is a highly disabling disorder prevalent worldwide, and is associated with increased mortality, poor quality of life, and low recovery rates. Over the past decade, there has been a great upsurge in the interest shown in studies focusing on EI in schizophrenia and related psychotic disorders. In this article, we briefly review the vastly expanding literature in this field, and focus on the rationale, the populations of interest, and the timing of EI approaches in psychotic disorders; we then review the approaches to EI with a view to identify evidence-based data. Finally, we will discuss the opportunities and challenges inherent in translating these models of care to mental health services in the Indian context.

### Rationale for early intervention in psychoses

The rationale for EI has been largely derived from two lines of evidence. First, several studies have documented an inverse relationship between the duration of untreated psychosis (DUP) and outcome of schizophrenia. In a meta-analysis[1] and a systematic review[2] longer DUP was associated with poorer response to antipsychotic treatment on several measures. Similar observations have been reported in the Indian context.[3] A recent meta analysis of five studies examining the association between DUP outcomes in the low- and middle-income countries found a significant negative correlation between DUP and improvement in symptoms after treatment.[4] DUP, which reflects treatment delay, may be potentially reduced by early detection and intervention in developing countries. In a recent population-based study[5] of patients with schizophrenia in a rural south Indian community, nearly two-thirds were not receiving medications, and the disability was significantly less in those who were receiving treatment. This highlights the importance of EI in the Indian setting.

Second, neuroimaging studies, using Magnetic Resonance Imaging (MRI), have observed a relationship between prolonged untreated illness duration, pronounced loss of gray matter volumes, and enlarged cerebral ventricles in patients with schizophrenia.[6] First-episode patients also have less prominent structural brain abnormalities than chronically ill patients. As a more direct evidence of illness progression, prospective follow-up studies of first-episode patients suggest continued loss of gray matter during the first few years after the onset of psychosis.[7] However, not all studies report a relation between DUP and brain morphological deficits from a series of cross-sectional neuroimaging studies.[8] In a unique study from South India, increased ventricular size, basal ganglia abnormalities, and involuntary movements were seen in chronic, never-treated schizophrenia patients.[9] Clearly more data are needed on this important question in the setting of Asian countries, such as India.

As the first episode of schizophrenia is frequently preceded by subtle psychotic-like symptoms and social withdrawal (the prodromal phase), one wonders whether the structural brain changes may emerge in parallel to the functional decline that characterizes the pre-psychotic period. Using a prospective study, Pantelis *et al*., (2003)[10] followed up MRI scans of 75 prodromal individuals, 23 of whom developed psychosis. Those who later became psychotic had less gray matter in the right medial temporal, lateral temporal, inferior frontal cortex, and in the cingulate cortex, bilaterally. This suggests that an active disease process may be taking place in the brain during the transition to psychosis. Indeed, it has been argued that continued untreated psychosis may be toxic to the brain.[11] If this is true, EI could halt or slow the progression of brain abnormalities in schizophrenia and the related psychoses. Additionally, postponement of the development of psychotic symptoms may allow the individual to have more time to establish a relatively more stable personality structure and ego functioning. The later illness onset in females may thus account for the better prognostic outcomes in women with the illness.

### Timing of early intervention

Schizophrenia typically begins in late adolescence or early adulthood, but its seeds are planted early in a long-term neurodevelopmental process eventually leading to deviant brain functioning. It is also evident that multiple and sequential etiological factors may interactively and additively contribute to the emergence of the illness. This view suggests that prevention and intervention need to be tailored to the stage of evolution of the disease processes in individuals predisposed to the disorder. The identification of risk factors and symptomatic indicators is critical for accurately selecting the at-risk people most appropriate for preventive treatment.

The early course of schizophrenia is punctuated by a premorbid phase (characterized by subtle cognitive and social difficulties), a prodromal phase (gradual beginning of subtle psychotic-like symptoms, social withdrawal and functional decline), the psychotic phase (with florid symptoms such as hallucinations and delusions), the transitional or recovery phase (a return to functioning, but with increased proneness to relapses and comorbid difficulties), and the stable or residual phase (with persistent cognitive and social deficits).

**Figure 1:**
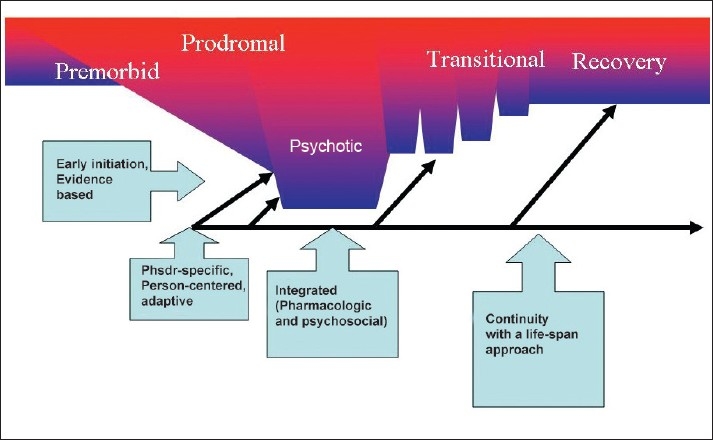
Key elements (EPIC model) of early intervention in schizophrenic illness

These observations point to the need to tailor treatments to the specific phases of the illness in which the patients present to us. There has been an increasing emphasis in recent years on *phase-specific* treatment approaches [Figure 1]. In the premorbid phase, the primary goal is early recognition of the illness; in the prodromal phase, the goal is reduction of the duration of untreated illness. In the psychotic phase, the primary goal is to reduce the overall duration of psychosis and harm prevention. In the transitional phase, key steps include reduction of secondary morbidity (such as depression and substance abuse) and relapse prevention using maintenance treatment with minimal side effects. Finally, the major goal in the stable phase is rehabilitation and restitution of the accruing cognitive and social deficits. McGorry *et al*.,[12] have proposed a clinical staging system, which provides a framework for earlier and safer interventions in a longitudinal evolution of schizophrenia from pre-psychotic to chronic phases.

### Approaches to early intervention

Pre-psychotic phase: The advent of atypical antipsychotic drugs has raised the hope that improvement in schizophrenia may extend beyond merely the control of psychosis, as some improvements in cognitive impairments may also occur, albeit modest.[13] Controlled trials of antipsychotics in the prodromal phase are relatively few.[14] A phase-specific intervention (low-dose risperidone and cognitive behavioral therapy) in people with prodromal symptoms lead to lower rates of conversion to psychosis at a six-month follow-up; this effect was not significant at the 12-month follow up. A US double blind study of the prodromal phase randomized patients to olanzapine or placebo.[15] Patients treated with placebo had non-significantly higher rates of conversion to psychosis and symptom severity by one year. Newly diagnosed patients with schizotypal personality disorder randomized to integrated treatments had lower rates of conversion to psychosis than those undergoing standard treatment.[16] Cognitive therapy also produced a significant reduction in transition rates in a British randomized control trial of ultra high risk subjects.[17]

Medications other than antipsychotics may also be worth considering in the prodromal phase, as part of the early intervention in the prodrome. A recent controlled study from Australia suggests that omega-3 fatty acids may be effective in preventing psychosis among individuals deemed to be at clinical risk for schizophrenia.[18] As cognitive deficits are among the earliest symptoms, pharmacological interventions targeting neurocognitive deficits may be beneficial; antidepressant medications were demonstrated to be effective, with fewer side effects, than antipsychotic medications, for prodromal individuals.[19] Overall, the results of EI in the pre-psychotic phase are encouraging; although the early studies are in need of replication.

Psychotic Phase: Are EI models of care effective in reducing long-term morbidity and disability in the early course schizophrenia after psychosis onset First-episode patients are in general more responsive to treatment than their chronic counterparts, but they are also more susceptible to adverse events.[20] The efficacy and relatively better tolerability of the newer atypical antipsychotics allows them to be used successfully in the long-term management of schizophrenia symptoms from the onset of illness. However, recent effectiveness studies have not found substantive differences in benefits versus risks of atypical compared to typical antipsychotics.[21,22]

Psychosocial treatments also work better early in the illness, perhaps because of the better neural plasticity that characterizes early phases of the illness. Cognitive remediation studies, using novel approaches to improve attention, memory, and social skills, have provided encouraging results with regard to improvements in the functional outcome, early in the course of schizophrenia.[23] Well-designed clinical trials are needed to further investigate these questions.

A large Scandinavian study (n = 547) compared the outcome in the first episode of schizophrenia randomized to either integrated treatment (assertive community treatment plus family therapy, social skills training, and medications) or standard care. The global and symptomatic outcome was better in the integrated treatment group by one year.[24] A Dutch study showed a superior outcome early in follow-up in first episode patients, but the relapse rates were not different when followed up five years later.[25] Overall, the results of the available controlled trials suggest that EI programs are encouraging, but are not conclusive;[26] initial effects may be beneficial, especially with *integrated* treatments, but are unlikely to be sustained over longer periods of follow-up. Thus, in order to reduce long-term disability, EI efforts should have seamless *continuity* with programs that primarily address the needs of chronic, relapsing patients.

### Research in early psychoses in India

Several early studies suggested that the outcome of schizophrenia may be more favorable in developing countries, such as India, compared to developed countries.[27] Consistent with this view, the Madras longitudinal study, one of the few long-term follow-up studies from the developing world, suggested that the course and functioning of schizophrenia were better than those found in many such studies from the developed nations.[28] However, in recent years this widely held view has been questioned.[29,30] The higher mortality observed among schizophrenia patients than among the general population in developing countries could at least, in part, account for a better outcome by selecting against the more severe forms of this illness.[31] It is also possible that the differences in prevalence of illicit drug use in schizophrenia across cultures may account for the variations in outcome.[31]

If patients with schizophrenia in the Indian setting are likely to have a better outcome, early interventions are expected to have more favorable outcomes. However, literature on this topic is relatively sparse. The feasibility, acceptability, and impact of services for the care of people with early phases of psychotic disorders in low-resource countries have not been researched well. Chatterjee *et al*.,[32] followed up 256 patients with early course psychotic disorders for a median four years, in a rural Indian community in Goa, receiving community-based intervention including psychotropic medications, psychoeducation, adherence management, psychosocial rehabilitation, and support. A vast majority (83%) were engaged with the program. Better outcome was predicted through higher education, lower baseline disability scores, family engagement, medication adherence, and participation in a self-help group. In a longitudinal study of schizophrenia patients in rural south India, Thirthalli *et al*., compared the outcome in patients who did versus did not receive treatment, and found that the disability was clearly better in those who received treatment.[33] In a controlled study, Kulhara *et al*.,[34] showed that structured psychoeducational intervention was significantly better than routine outpatient care on several outcome indices. Early interventions are therefore feasible and acceptable in low-resource settings. However, controlled studies are very few, and are needed to determine the effectiveness of such interventions in early course psychosis patients.

### Challenges to EI programs in India

Several roadblocks exist that may constrain the application of EI models in the Indian setting. Challenges that beset early psychosis interventions in India stem from those that affect mental health care in general. While the mental health scenario is improving in the country, constraints to access availability and affordability of care continue.[35–37] Manpower resources remain unequal between the rural[38] and urban[39–41] areas; funding, number of trained mental health professionals, inpatient, emergency and crisis facilities, and psychotropic medications are in short supply.[42] Psychiatric care is delivered through the mental hospitals, psychiatric departments of teaching general hospitals, district hospitals, and district hospital mental health centers, as part of the National Mental Health Program. Mental health care is integrated with general health care[43,44] and offered to rural areas through a network of primary health centers,[45] which have a well-placed referral system to district mental health programs for severe cases.[46–48] Specialty care, on the other hand, is present only in a few centers of repute and program-based care (such as EI for psychoses) is rare. The private sector, which provides the mental health care delivery system, is completely isolated from the government delivered care.[49] Quality assurance, guidelines for evidence-based practice,[50,51] and comprehensive community care based on the multidisciplinary concept remain undeveloped.[52] Not surprisingly, responsibilities of community care, follow-up, compliance, risk management, and cost of care, particularly in the private sector, continues to be borne by the families.[53,54]

Lack of awareness of the illness and of services is a major roadblock. In a study on mental health literacy in a rural community in Maharashtra, using case vignettes of subjects with mental illness, most respondents were aware of mental illnesses such as depression, but awareness of mental health treatments and psychiatric medications was negligible.[55,56] Stigma is an important roadblock to seeking help, and has complex cultural determinants.[57,58] There are differences between rural and urban schizophrenia patients: urban patients feel the need to hide their illness and avoid illness histories in job applications, while rural patients experience more ridicule, shame, and discrimination. Non-medical belief models that influence patterns of health care seeking are also important. A study in Vellore[58] observed that a majority of schizophrenia patients (70%) considered spiritual and mystical factors as the cause of their illness; 22% held multiple models of illness. Those who held a biomedical concept of the disease, predictably, had a better insight compared to those who held non-medical beliefs. Psychoeducation efforts should therefore focus on imparting evidence-based knowledge about the illnesses in a culturally acceptable manner.

### Early intervention in psychoses in the Indian context

By contrast to the challenges given earlier in the text, however, there are also positive trends that augur well for the potential success of EI programs in India. First, with increasing literacy and especially the widening use of the media and the internet, it should be possible to increase public awareness of early signs of mental illness and the importance of early intervention. The goals should be to fight stigma and to promote early identification and referral. Popular press and media are important allies in efforts to sensitize the general population.[59] The second important step is to train and educate primary health care providers and local family physicians,[60,61] as well as advocacy groups. The third key step is to accomplish continued, qualitative and practical education for the mental health professionals, which can be effective.[62–64] Establishing support groups and psychoeducation is the key to success. Networking and sensitization of the existing voluntary organizations in different social sectors is required. ‘Out-reach’ programs for the early detection of serious mental illness in special and vulnerable groups in schools and universities can potentially prevent the snowballing of untreated illnesses toward chronicity and dysfunction.[65] A potentially valuable and novel approach for EI in the Indian context is to train family members in principles of case management that works well in early intervention studies[24].

Wide-scale establishment of program-based care of ‘early intervention’ in the Indian community, however, will depend on policymakers increasing the priority for mental health among diverse aspects of health care, and incorporating EI elements in the National Mental Health Program, similar to efforts elsewhere, for example, Canada and Australia.[66,67] Thus therapeutic interventions need to be adapted to integrate biomedical illness models with culturally accepted models.[68]

There may be other challenges to EI that need to be further researched. First, many prodromal patients may remain undetected. A recent unpublished study by Thirthalli and colleagues in South India observed that only 7% of schizophrenia patients out of 300 had a discernable prodrome/pre-psychotic phase. The researchers had interviewed family members who had lived with the patients for years, even before the onset of illness. It is possible that low levels of antecedent substance abuse may at least in part account for this. Tolerance in the Indian families for behavioral abnormality and/or disability from the illness may also be high; over 50% of symptomatic schizophrenia patients did not receive drugs, but nearly all lived with their families. Therefore, tolerance to the prodromal phase may be higher, to a level that it is often not recognized. Second, considering that a higher clinical skill is required for ‘diagnosing’ the prodrome, community health workers/ nurses may find it hard to make an accurate detection of these cases. Third, given the stigma of mental illness in India, early detection/identification of prodrome may lead to unnecessary negative social consequences to these individuals, many of whom may not even develop the illness eventually (false-positives). It is presently not known if the direct and indirect costs of EI, including the side effects, do not overweigh the benefits of such interventions. Clearly more compelling evidence is needed from relevant populations before a policy on EI can be made for India. Finally, a critical need is data on real-world effectiveness of EI initiatives in the Indian context. Questions to be addressed include: What does EI cost, and who will pay for it? Are EI approaches practical, easy to teach and measure? Will EI reduce health care costs in the long run? Do all relevant stakeholders ‘buy-in’ to the proposed models (public and private payers, family members, and consumers)?[69] Indian experience with chronic mental illness also suggests that community-based interventions may not be sustainable without involvement of the family and the community, with regular support from the mental health professionals regularly.[70] The EI initiatives may not be different.

## CONCLUSIONS

In summary, early interventions represent an important paradigm shift in the management of schizophrenia. For EI efforts to be successful, the following elements (which we summarize as the EPIC principles, see Figure 1) need to be present: Early Initiation and Proven Effectiveness, Phase-specific, Integrated (psychosocial and pharmacological), and Continuity of Implementation. EI programs will hopefully enhance the smooth transition between child/adolescent and adult services for serious psychotic illness. EI programs will offer a great opportunity to set in motion a life-cycle approach to management of these illnesses, and not just for the first two to three years.

The idea that early intervention will forestall long-term suffering is not unique to psychotic illness, but is well known for chronic diseases (such as diabetes and hypertension) across all of medicine. However, EI models of care have been criticized as being too elitist and their effectiveness unproven.[70] Therefore, to address such criticisms, more evidence-based data is urgently needed, especially in developing countries such as India, so that increased allocation of the already meager resources can be well justified.
